# A scientometric, visualization, and content analysis on surgical endodontics from inception to 2023

**DOI:** 10.1007/s10266-025-01114-4

**Published:** 2025-04-28

**Authors:** Violetta Chasoglou, Anastasios Katakidis, Anastasia Fardi, Konstantinos Kodonas, Nikolaos Economides

**Affiliations:** 1 Department of Endodontology, School of Dentistry, Aristotle University of Thessaloniki, Thessaloniki, Greece; 2 Department of Dentoalveolar Surgery, Surgical Implantology & Radiology, School of Dentistry, Aristotle University of Thessaloniki, Thessaloniki, Greece

**Keywords:** Apicoectomy, Bibliometrics, Scientometric analysis, Surgical endodontics, Periapical surgery

## Abstract

In persistent periapical lesions, periapical surgery may be considered an alternative treatment option to remove inflammatory and necrotic tissue from the periapical region, seal the apical root end, and promote tissue regeneration. The present scientometric analysis aims to analyze the scientific landscape in the field of surgical endodontics, identify research trends, and classify popular topics. A thorough literature search was conducted in the Web of Science and Scopus databases on 29 September 2023 to retrieve all articles related to surgical endodontics. The articles were analyzed with Biblioshiny software, and the following details were collected: publication titles & years, authors’ names and affiliations, journals’ metrics, citation counts, author keywords, the field of study, study design, and outcome. A total of 1988 publications concerning surgical endodontics from 1935 until September 2023 were identified and further analyzed. Sixty-two countries contributed to scientific production in this field and the USA was the most productive among them. Von Arx T. and the Journal of Endodontics was the most relevant author and journal, respectively. “Materials” was the most prevalent title term and “apicoectomy” was the most used keyword. The distribution of the scientific literature on surgical endodontics was as follows: 38.3% basic science, 15.8% reviews, 37.2% observational studies, and 8.7% clinical studies. Overall, 31% of the conducted research concerned the materials and 19% the techniques used in clinical practice. This bibliometric analysis describes the research landscape in surgical endodontics and converts the acquired data into useful information regarding the current and future trends of this field.

## Introduction

Root canal treatment is considered a predictable and reliable procedure with success rates ranging from 75 to 85% [1]. Despite the reported favorable outcomes, many cross-sectional studies have found that the prevalence of post-treatment disease may exceed 55% in root-filled teeth [2–4]. According to these findings, further intervention such as retreatment or periapical surgery may be necessary. The clinician should decide on the best treatment option considering various parameters such as the intervention site and the risks involved [5]. Despite advancements in non-surgical endodontics, periapical surgery remains a basic and sometimes unique treatment option for treating persistent periapical pathosis. The basic goal is to remove inflammatory and necrotic tissue from the periapical region, seal the apical end of the root canal, and promote tissue healing [6]. Recent clinical outcome studies have reported success rates ranging from 63 to 95% [7–9]. 

A wide variety of materials and techniques have been studied through laboratory research, evaluated in clinical studies, and finally used in clinical practice. Therefore, there is a wealth of publications and a vast amount of knowledge available on surgical endodontics demonstrating the continuous evolution of this field. Bibliometrics can be used for the scientific mapping of documentation while analyzing large volumes of data [10]. Bibliometric analysis may represent a viable tool to track the evolution of this research field over time, identify collaboration patterns, research trends, and gaps, and guide future research.

To the best of our knowledge, a scientometric analysis of the surgical endodontic literature has not been conducted so far. The purpose of the present study is to establish the scientometric landscape of the field of surgical endodontics from its inception to September 2023. Specifically, this bibliometric analysis aims to (i) provide valuable insights into research trends by analyzing the most common title terms and author keywords, (ii) classify popular research topics by subject area, (iii) highlight the most productive journals, the most contributing countries and the most influential authors involved in this scientific field, and (iv) provide a content analysis of the published research on surgical endodontics.

## Materials and methods

**Fig. 1:**
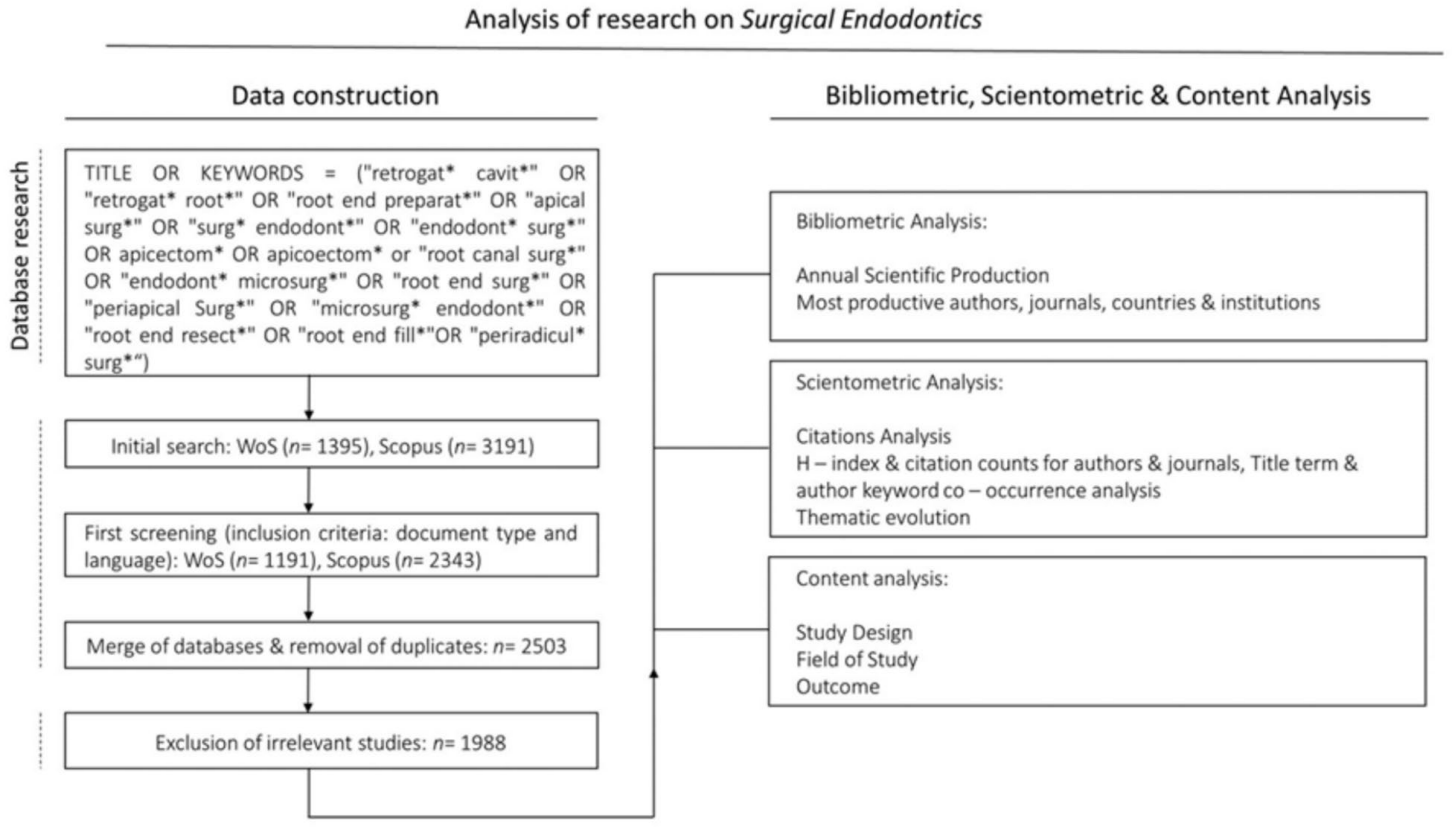
Search and analysis strategy

The present bibliometric analysis followed the preliminary guidelines for reporting bibliometric reviews of biomedical literature (BIBLIO) [11]. On September 29, 2023, a scientometric analysis of literature related to surgical endodontics was conducted, using the Web of Science (WoS) by Clarivate Analytics and Scopus maintained by Elsevier. WoS and Scopus are fundamental tools for dental researchers, offering the necessary resources to conduct thorough literature reviews and impactful bibliometric studies. Both databases have extensive collections of peer-reviewed indexed journals and provide comprehensive access to citation data, making them suitable for assessing research output, collaboration patterns, and identifying influential authors and works [12, 13]. The search involved specific descriptors in the “title” term and “author keyword” query following the search strategy outlined in Fig. 1. The search results were first screened for document type and language. Only original articles and review papers published in English were included for further analysis. Meeting abstracts, editorials, notes, books, proceedings, early access, and non-English publications were excluded.

RStudio package (version 2023.12.1 + 402) was utilized to combine, edit, and merge bibliographic data from the databases identifying duplicates and consolidating the results into a single file format containing 2.503 publications. A bibliographic data frame was created, including all selected documents with details, such as: publication titles, years, authors’ names and affiliations, journals’ metrics, citation counts, and author keywords. All included publication abstracts were screened by two independent researchers (V.C. and A.K.) to exclude documents irrelevant to periapical surgery. In cases of discrepancy, a third reviewer (K.K) was consulted. The eligible documents were further processed using the Biblioshiny, a web interface for Bibliometrix version 4.0, an open-source tool.

Complimentary analysis regarding field of study, design, and outcome analysis was performed. In cases where the field of study was not identified in the abstract, an in-depth search was conducted through the full text of the article. The analysis recorded the following bibliometric indicators:

- Annual scientific production.
- Most influential authors.
- Contributing countries, collaborative patterns, organizations, and journals.
- Research trends, title terms, and author keywords.
- Content analysis, study design, field of study, and outcome analysis.
- Citation analysis.

All data were analyzed and visualized by tables, charts, and diagrams. In thematic map analysis, the conceptual evolution of various themes was visualized in a diagram with clusters created from the co-word title term analysis divided them into four quadrants.

## Results

The initial search yielded 4.586 documents. After applying the inclusion and exclusion criteria, merging the bibliographic data, and removing the duplicates, the final sample for the bibliometric analysis consisted of 1.988 eligible publications dating from 1935 to September 2023.

### Annual scientific production

**Fig. 2:**
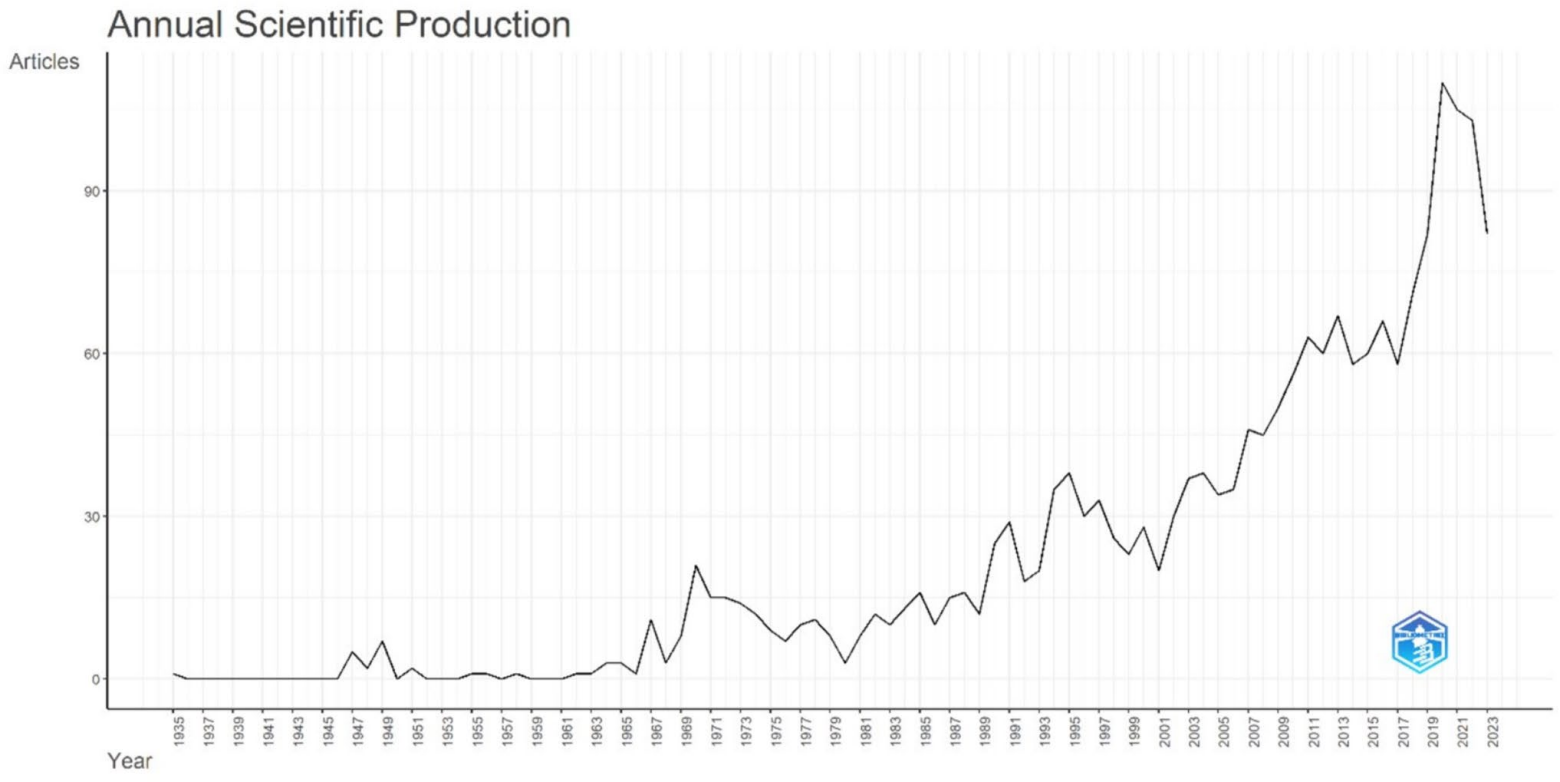
Annual scientific production

The overall scientific production rate has increased since 1935 with changes in productivity fluctuating from year to year (Fig. 2). In the initial stages, there was minimal but consistent research interest in the topic until 1970. During the next period and until 1994, a moderate increase in publications was observed (35). After 1995, researchers have continued to devote significant attention to surgical endodontics, leading to a notable increase in publication productivity. This trend continued until 2009, when the number of publications exceeded 50 per year. In the last stage from 2020 until now, an exponential growth in publications has been observed with the number of publications surpassing 100 per year. The oldest article published in 1935 performed histopathologic analysis on the root-end and the periapical tissues of treated maxillary incisors [14]. The most recent articles were a case report on the management of a cystic lesion perforating the lingual plate [15] and a case-series studying the use of a 3D-printed surgical guide about [16].

### Author analysis

A total of 4890 authors have contributed to the published research on periapical surgery. Among them, 12 authors wrote more than 20 papers, while 48 authored 10 or more publications. Additionally, there were 243 documents with a single author. The average number of co–authors per publication was 3.87. The most productive author was Von Arx T. with 66 publications, followed by Kim S, and Taschieri S. with 39 publications, each. Considering the number of total citations, Torabinejad M. ranked first with 30 publications and 5110 citations, followed by Kim S. (2360 citations), Von Arx T. (2340 citations), and Taschieri S. (1121 citations).

### Countries, organizations and journal analysis

**Fig. 3:**
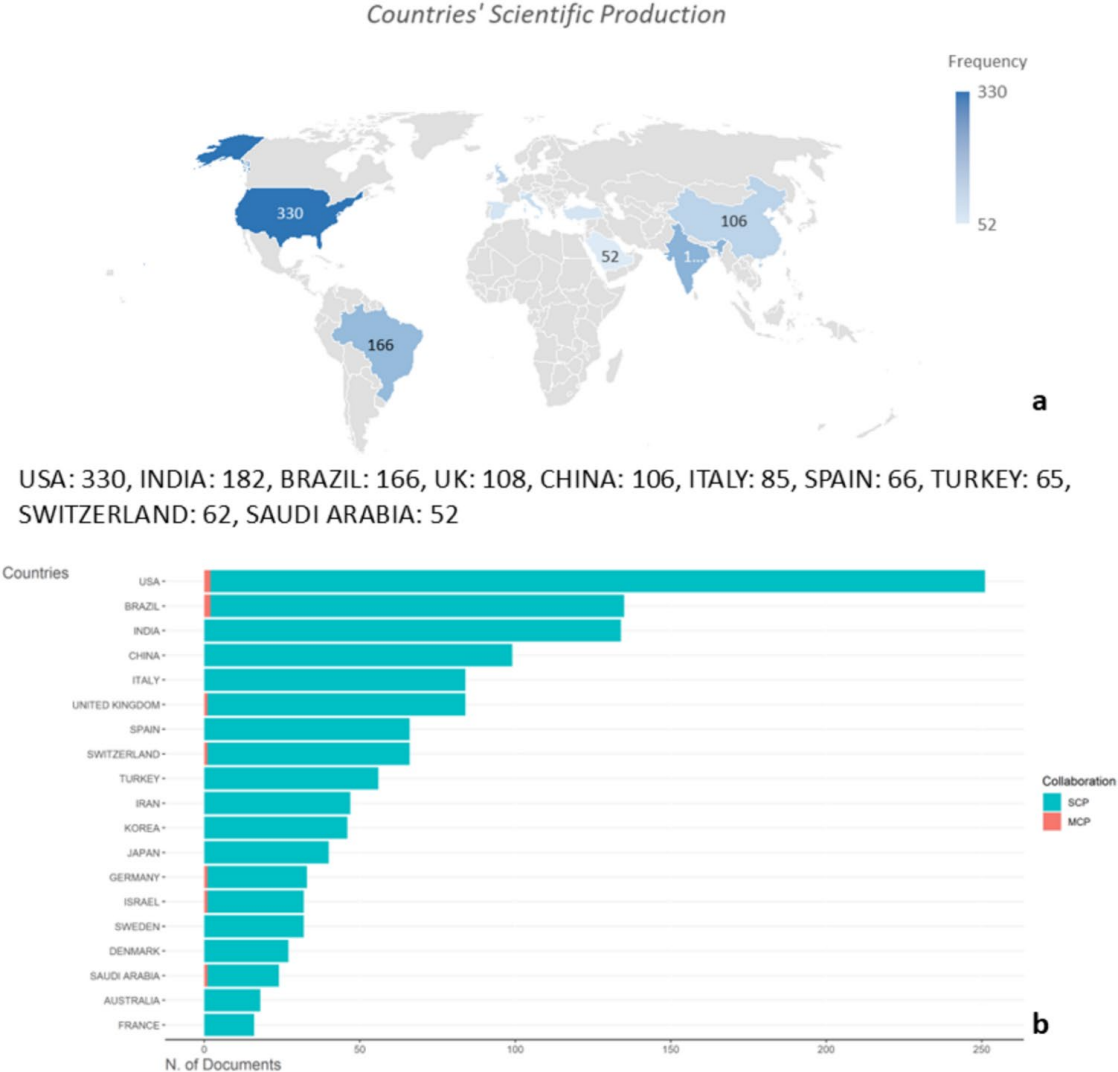
**A** Countries’ scientific production. **b** Corresponding authors’ countries, *SCP* single country publications, *MCP* multiple country publications

A total of 62 countries contributed to surgical endodontics. The USA was the leading country with a total of 330 publications (Fig. 3a), followed by India and Brazil with 182 and 166 published documents, respectively.

Considering the corresponding author’s country, the USA led again the ranking with 251 documents, followed distantly by Brazil (*n* = 135), India (*n* = 134), and China (*n* = 99) (Fig. 3b). The University of Bern in Switzerland published the highest number of documents, followed by the University of Pennsylvania and the Loma Linda. It is worth noting that most publications resulted from authors affiliated with a single country. Only 13 publications were the result of international collaborations. The USA received the highest number of citations (*n* = 9152) followed by Switzerland and Italy with a total of 2942 and 2874 citations, respectively.

Overall, 391 journals published 1988 manuscripts. More than half of the literature (*n* = 1060) was published in 20 journals. The Journal of Endodontics appeared in the first place with 416 articles, followed by the International Endodontic Journal (*n* = 183), and Oral Surgery, Oral Medicine, Oral Pathology, Oral Radiology, and Endodontology (*n* = 134). Among the journals with the highest production, the first two also had the highest impact factor and received 19,584 citations (h-index 68) and 5233 citations (h-index 43), respectively.

### Keyword co-occurrence analysis and thematic map evolution

**Fig. 4:**
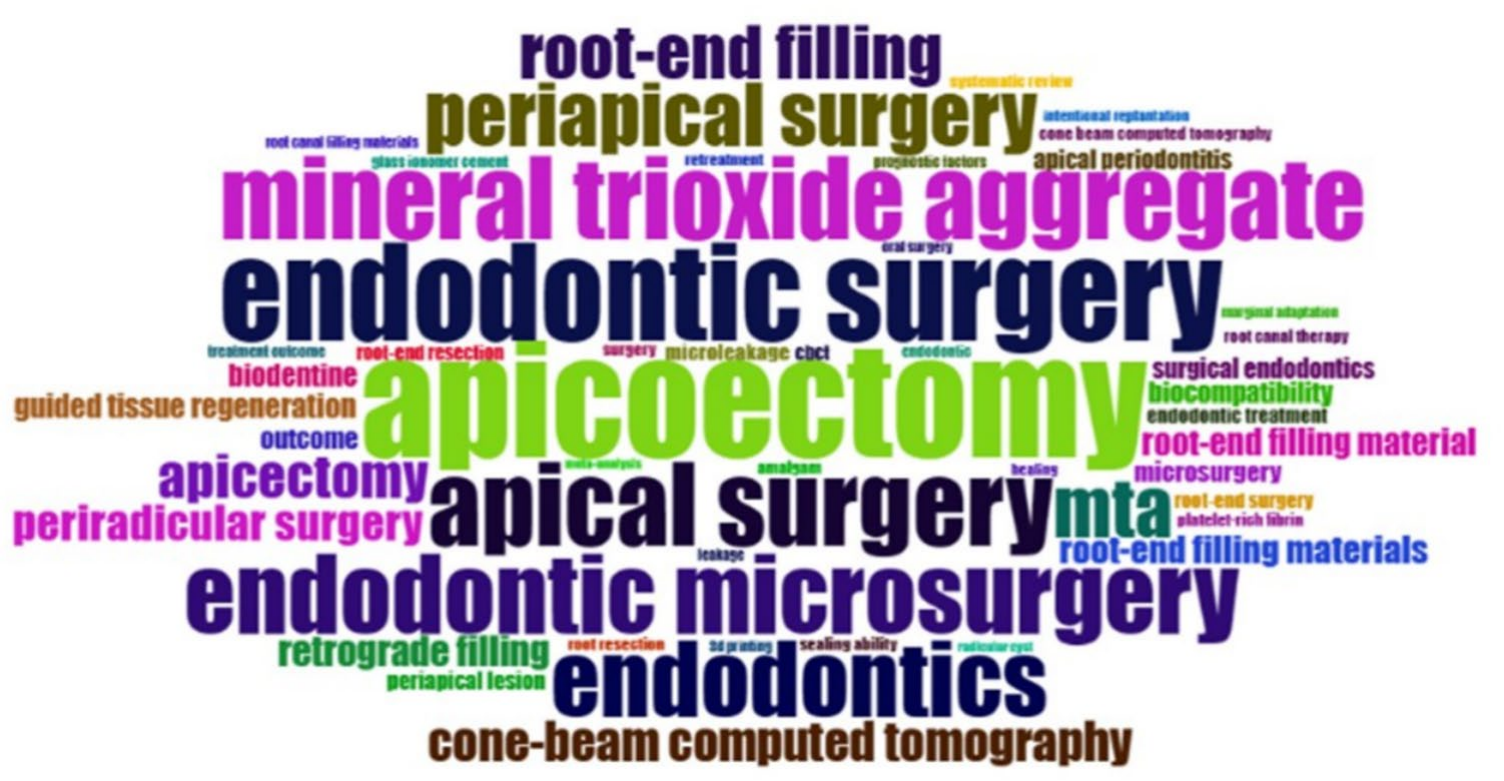
Word cloud based on author keywords. The different colors represent various keywords while the difference in font size indicates their frequency

**Fig. 5:**
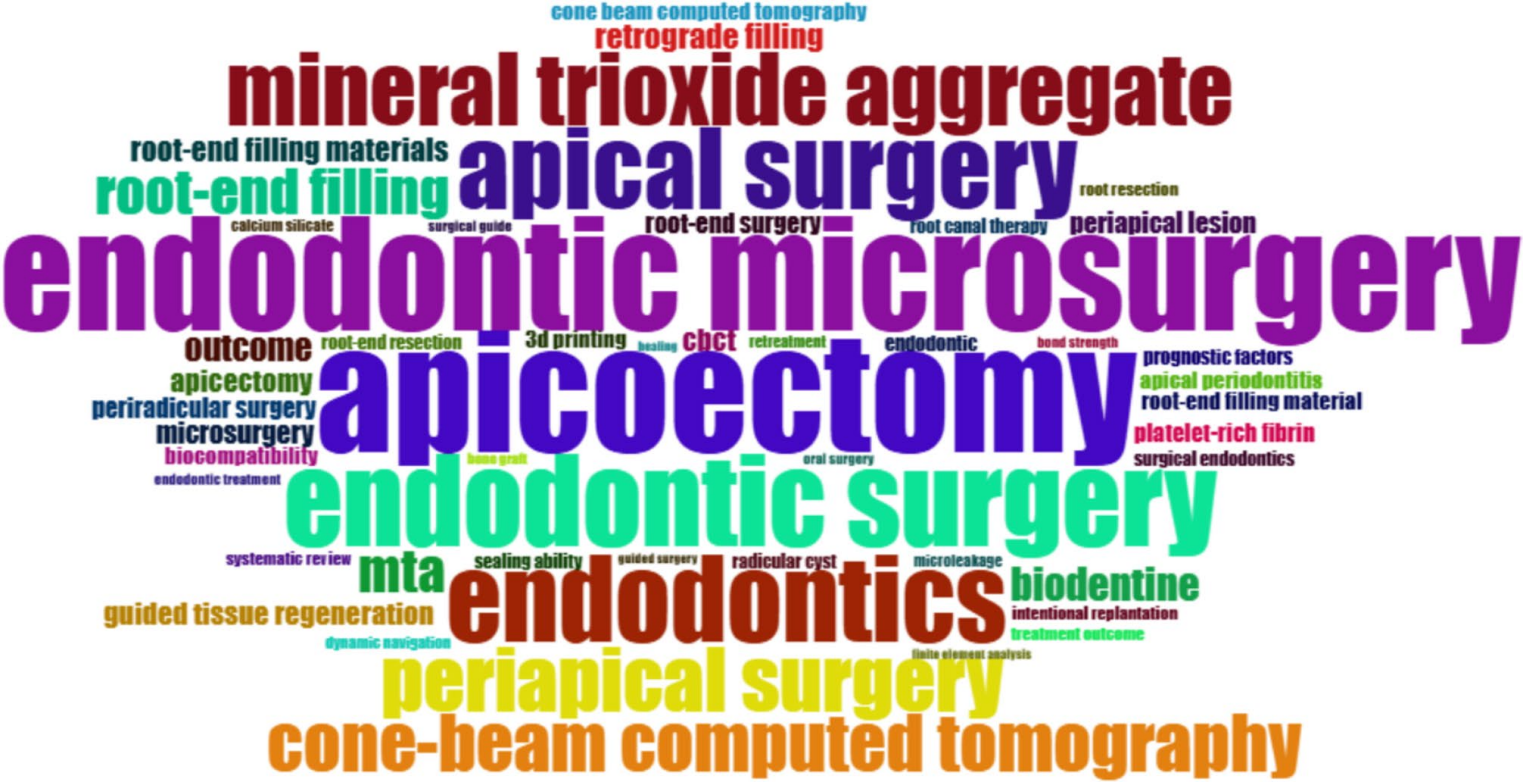
Word cloud based on author keywords in the years 2014–2023

A topic keyword analysis revealed the most prevalent topics and issues within the research field. A visual representation of the 2197 author keywords extracted from the included documents can be seen in Fig. 4. The different colors represent various keywords, while the difference in font size indicates their frequency. “Apicoectomy” is the most frequent keyword with 168 occurrences. Without considering the descriptors used for the search strategy, “mineral trioxide aggregate” (*n* = 119) with its acronym “MTA” (*n* = 86) and “cone-beam computed tomography” (*n* = 57) may be identified as prominent areas of research or research hotspots within the discipline of surgical endodontics. A topic keyword analysis for the years 2014–2023 revealed that “endodontic microsurgery” was the most frequently used keyword (Fig. 5). “Mineral trioxide aggregate” and “cone-beam computed tomography” were two of the most frequent keywords used even in this time frame.

**Fig. 6:**
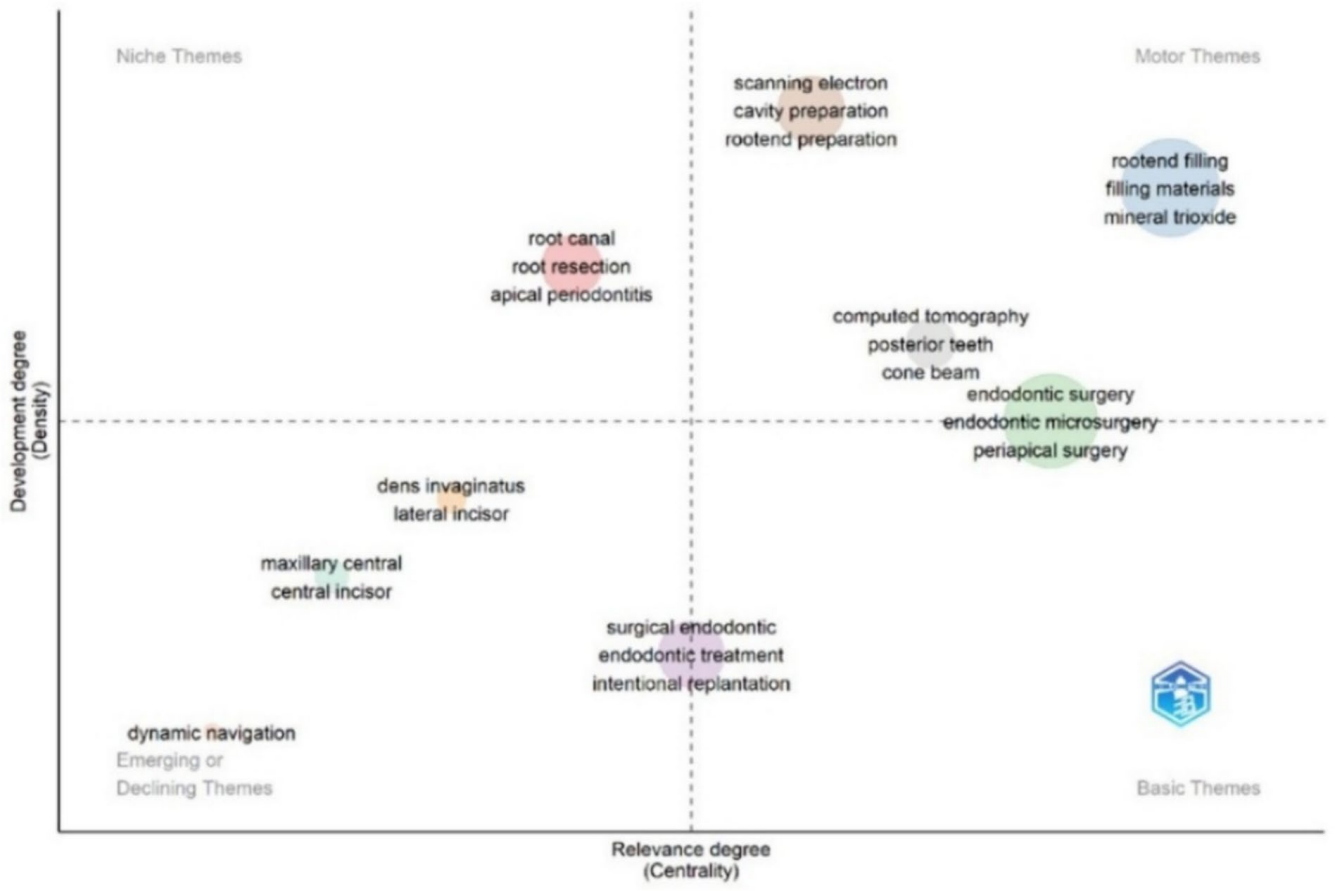
Thematic map analysis based on title terms. In a strategic diagram, each theme is defined by two parameters, density and centrality. Based on the values of these parameters, themes are placed in the corresponding quadrant(s). The top right quadrant contains well-developed motor themes, and the top left quadrant includes highly developed and isolated themes. Weakly developed, either emerging or disappearing, marginal themes are included in the bottom left quadrant, while important themes are presented in the bottom right quadrant

As shown in Fig. 6, a title term thematic map was created to identify research directions and themes, and present their evolution. Three clusters on the first quadrant (top right) are mainly focused on “root-end filling materials and mineral trioxide”, “scanning electron, cavity and root-end preparation”, and “computed tomography, posterior teeth and cone beam”. The cluster of “endodontic surgery, endodontic microsurgery and periapical surgery” was identified as motor and basic theme. Similarly, the cluster of “surgical endodontic, endodontic treatment and intentional replantation” was recognized as a basic and declining theme. “Dynamic navigation, maxillary central, central incisors, dens invaginatus and lateral incisors” are three independent clusters belonging to the same area of emerging themes. The cluster of “root canal, root resection and apical periodontitis” corresponds to highly developed and isolated themes in the main area being considered as niche themes.

### Content analysis

#### Study design

Regarding the document type of the included publications, 1749 original scientific articles and 327 reviews were identified. From the original articles, there were 795 basic research, 773 observational studies, and 181 clinical studies of which 132 were randomized clinical trials (RCTs) and 49 were non-RCTs. Specifically, in vitro studies made up approximately one-third of the total sample (27%), while case reports ranked second (17%). Prospective or retrospective cohort studies represented 12%, while RCT accounted for 6% of the published papers. The percentage of reviews published, including narrative, systematic, or meta-analysis, totaled 14%.

### Field of study

Approximately one-third of the analyzed studies (31%) focused on material research, 19% investigated techniques used in clinical practice, and 14% of the studies evaluated outcome. Case reports accounted for 10% of the published studies. Other fields of study in smaller numbers considered the impact of various factors on treatment plan development (5%) or potential prognostic factors in surgical endodontics or causes of failure (4%). Histological studies made up 5% of the included publications. Many studies had more than one field examined, with the most common combination that of success–failure evaluation and type of incision technique.

A complementary subdivision analysis was performed for materials, techniques, and outcomes that represented the top three study fields. Analysis of studies evaluating surgical endodontic materials revealed that sealing ability (27%), type of grafting materials together with GTR techniques (17%), and biocompatibility/cytotoxicity assays (11%) were the main topics addressed. Less commonly examined characteristics involved the influence of the materials on pH and ion release (8%), bioactivity (6%), as well as marginal or interfacial material adaptation (6%).

Regarding periapical surgery techniques, most studies focused on apical cavity preparation (18%), the use of ultrasonics (17%), and evaluation of parameters related to apical resection length and angle or different types of instruments (15%). The use of navigation systems was examined in 14% of the publications. Less commonly studied topics included the use of lasers (11%), the impact of enhanced visualization (5%), and various aspects of the surgical procedure, such as osteotomy, flap design, incision techniques, anesthesia, and hemostasis.

The third largest field of study group focused on evaluating outcomes with 35% of studies primarily assessing the materials used and 24% of them the predisposing factors. Outcome has been shown to be related to the type of tooth investigated (anterior posterior), the follow-up period, and the success criteria utilized in each study. 14% of the articles studied the outcome in molars, 4% in posterior teeth (premolars and molars), and 50% of them examined tooth type as a possible prognostic factor. Outcome in these clinical studies was mostly evaluated through clinical examination and the radiographic criteria of Rud and Molven (71%). The follow-up period ranged from 6 months to 23.5 years and the overall success rate from 44 to 93.1%. Nearly three-quarters of the clinical studies that performed statistical analysis mentioned that there was no significant difference in the success rate between the various tooth types. From the studies that found a significant difference, 84.6% of them mentioned a more favorable outcome for anterior teeth.

### Citation analysis

A citation analysis was performed to investigate the most important documents. The top 10 most cited articles on surgical endodontics in the years 2014–2023, based on data from the Web of Science database, are presented in Table 1. Nearly half of the top 10 most cited papers centered around the characteristics of calcium silicate cements. Three studies explored 3D printing and imaging applications in clinical practices, such as guided endodontics, while another study compared 2D and 3D healing assessment. Additionally, a case report examined the osteo-inductive growth factors for enhancing bone regeneration and a systematic review and meta-analysis investigated the management of failed root canal treatments through retreatment or apical surgery.

**Table 1:** Top 10 most cited papers in the years 2014–2023

Publication	No of citations
Prati C, Gandolfi MG. Calcium silicate bioactive cements: biological perspectives and clinical applications. Dent Mater. 2015;31:351–70	320
Chen I, Karabucak B, Wang C, Wang HG, Koyama E, Kohli MR, Nah HD, Kim S. Healing after root-end microsurgery by using mineral trioxide aggregate and a new calcium silicate-based bioceramic material as root-end filling materials in dogs. J Endod. 2015;41:389–99	96
Del Fabbro M, Corbella S, Sequeira-Byron P, Tsesis I, Rosen E, Lolato A, Taschieri S. Endodontic procedures for retreatment of periapical lesions. Cochrane Database Syst Rev. 2016;10:CD005511	87
Gandolfi MG, Siboni F, Primus CM, Prati C. Ion release, porosity, solubility, and bioactivity of MTA plus tricalcium silicate. J Endod. 2014;40:1632–7	86
Sureshbabu NM, Selvarasu K, V JK, Nandakumar M, Selvam D. Concentrated growth factors as an ingenious biomaterial in regeneration of bony defects after periapical surgery: a report of two cases. Case Rep Dent. 2019;2019:7,046,203	81
Anderson J, Wealleans J, Ray J. Endodontic applications of 3D printing. Int Endod J. 2018;51:1005–18	81
Giacomino CM, Ray JJ, Wealleans JA. Targeted endodontic microsurgery: a novel approach to anatomically challenging scenarios using 3-dimensional-printed guides and trephine burs-a report of 3 cases. J Endod. 2018;44:671–77	74
Strbac GD, Schnappauf A, Giannis K, Moritz A, Ulm C. Guided modern endodontic surgery: a novel approach for guided osteotomy and root resection. J Endod. 2017;43:496–501	74
Butt N, Talwar S, Chaudhry S, Nawal RR, Yadav S, Bali A. Comparison of physical and mechanical properties of mineral trioxide aggregate and biodentine. Indian J Dent Res. 2014;25:692–7	69
Schloss T, Sonntag D, Kohli MR, Setzer FC. A comparison of 2- and 3-dimensional healing assessment after endodontic surgery using cone-beam computed tomographic volumes or periapical radiographs. J Endod. 2017;43:1072–9	68

## Discussion

Bibliometric analysis is utilized to obtain quantitative and qualitative insights into the development of a research field employing descriptive statistical methods. Specifically, bibliometrics is utilized to assess research output, analyze trends, and measure impact, attribution, and distribution of scientific papers, journals, or countries [17]. Scientometrics is a method for quantitatively evaluating performance and mapping analysis of scientific productivity. It explores communal relationships among publications and authors’ cooperations, both in general research fields and specific disciplines using statistical and visualization techniques [17]. The information extracted from the analysis is valuable for researchers to discover research trends, identify research gaps, and direct future research strategies and interest on demanding and uncharted fields.

This bibliometric, scientometric, and content analysis aimed to provide an overview of the existing scientific publications under the theme of surgical endodontics from inception until September 2023 combining the content of two major databases. The productivity ratio and impact of authors, journals, and countries were evaluated using various bibliometric indicators (annual scientific production, influential authors, countries, collaborative patterns, organizations and journals, research trends, title terms, and citation analysis). More specifically, the overall scientific production rate has increased since 1935 with characteristic peaks observed in 1995, during the period 2010–2015 and recently in 2021. Over the span of 88 years, a total of 1988 research articles were published across 391 journals with the annual growth rate of publications being estimated at 5.14% per year. These articles were authored by 4890 individuals from 1832 institutions, and 64 countries. Most articles were written by authors from the United States. Most of the evidence was published in the two highest impact factor journals in the field of endodontics, the *Journal of Endodontics* and the *International Endodontic Journal*.

Interestingly, authors’ productivity in terms of publication numbers did not directly correlate with the impact expressed by citations. Torabinejad M. emerged as the most impactful author in surgical endodontics with 5110 citations, despite being ranked as the 7th most relevant author with 30 publications. It becomes more than obvious that evaluating an author’s impact based solely on the total number of publications may not provide an accurate assessment. The invention of mineral trioxide aggregate (MTA) by Torabinejad M. in 1993 revolutionized endodontic practice and periapical surgery leading to a significant improvement in the success rates worldwide, a fact highlighted by the citation count of the author. On the other hand, the use of the total number of citations for the evaluation of a researcher’s impact has limitations too. Factors such as the type of article (reviews tend to receive more citations than original papers) [18], publication years (older articles gain more citations than recent ones), and databases used may influence citation counts [19]. Consequently, the actual impact of the author cannot be evaluated by simple (total number of publications and citations) or more complex bibliometric indicators (Lotka’s Law, h-index, etc.), as the versatile nature of bibliometric analyses calls for a multifactorial approach and interpretation.

The main subject areas with high degree of development and relevance in the field of surgical endodontics were identified through different bibliometric mapping methods. These subject areas were categorized and clustered as themes using the author keywords and title terms. As evidenced by thematic map and co-occurrence analysis, research topics were not limited to materials, techniques, and outcomes, but also encompassed the fields of microsurgery and CBCT. The absence of clusters in the basic theme quadrants highlights the need for further research in this specific area. Dynamic navigation” is a minimally explored emerging theme, examined in 14% of the publications. Future research and systematization efforts should be encouraged. Anterior teeth, including those with developmental disorders, represent themes of low centrality and density, indicating that further investigation is not necessary.

When evaluating literature, the most important aspect to validate the power of included studies is to analyze their study design. Though it is crucial for evidence-based practice, it is also mandatory that the vast amount of available knowledge should be condensed and integrated into recommendations that clinicians can easily access and apply to meet the needs of their patients. Based on the findings of the current scientometric analysis, nearly half of the studies were classified as either in vitro experiments or case reports. This suggests that a significant portion of the published data falls within the lowest level of the evidence hierarchy. On the contrary, high evidence clinical trials and systematic reviews represent only 6% and 4% of published papers, respectively. Though case reports represent the first step to disseminate knowledge of rare diseases or new innovative techniques, and laboratory research is a prerequisite for future preclinical and clinical evaluation of the biological and physicochemical properties of new materials, there is still a need for more high-level evidenced research on surgical endodontics. Perhaps, this is a reason for the variable range of outcome results. On the same time, the advancement of techniques and materials employed in clinical practice makes it difficult to perform a direct comparison as is mentioned in various clinical studies with long follow-up periods [20].

Material research is a popular field of study in surgical endodontics, as indicated by thematic map and content analysis. Interestingly, this point was also emphasized in an analysis of evolving endodontic research trends that examined the differences in the content of the two leading endodontic journals over a 10-year period [21]. The number of material-related publications peaked around 1991. At that time, most of the papers evaluated the properties of amalgam, glass ionomer cement, and composites. Despite the limited number of materials traditionally used in surgical endodontics, the discovery of MTA and its potential benefits made a significant breakthrough. The greatest advantage of MTA was based on its bio-inductive properties as evidenced by histological studies showing true tissue regeneration [22]. Between 1993 and 1995, five of the ten most cited MTA-related documents were published [23–27]. All of them evaluated the properties of MTA and were authored by Torabinejad M., who was the most impactful authors acquiring more than 5000 citations in total. This peak in citations also coincides with a peak in the annual scientific production chart around the same years (1993–1995) highlighting the impact of these documents on the research and clinical field. During the following years, another peak was observed around 2013 with studies evaluating the properties of new tricalcium silicate materials. These bioactive materials can induce periapical healing as they interact with surrounding tissue fluids to promote the formation of calcium hydroxide and calcium phosphate compounds [28, 29].

Besides materials, new techniques and instruments have been evaluated by almost half of the studies included. In 1997, when new access armamentarium (piezo tips, ultrasonics, and micro-instruments) has been introduced, the root resection bevel angle was set from 45^0^ to 0° or 10°. According to the data presented in this analysis, a new evolving area of research has emerged during the same period concerning the use of magnification devices. Therefore, several systematic reviews and meta-analyses have been conducted to evaluate enhanced visualization in clinical practice and its impact on surgical endodontics outcomes [30–32]. These changes along with the use of magnification confirmed the transition from traditional root-end surgery to apical microsurgery.

The trend toward endodontic microsurgery is supported by the findings of keyword analysis and the 10 top cited list for the period 2014–2023, as shown in Fig. 5 and Table 1. Authors and researchers frequently use this keyword across various fields of study. Analysis of the top 10 most cited papers during this period highlights the ongoing interest in materials used in endodontic microsurgery, particularly the introduction and advancements of calcium silicate cements and osteo-inductive growth factors to optimize healing. The use of 3D surgical guides for targeted microsurgical procedures improved accuracy and efficiency, allowing for less traumatic interventions. The integration of 3D imaging in dentistry and surgical endodontics has improved treatment planning and surgical procedure by enhancing visualization of the surgical field. This advancement has raised questions about the outcomes of surgical endodontics in terms of healing and bone regeneration compared to periapical radiography. Finally, within the limited evidence provided by a Cochrane systematic review, only the use of ultrasonics was shown to significantly enhance clinical and radiographic healing outcomes. It is noteworthy that the majority of the top 10 cited papers have study designs at the lowest level of scientific evidence (8 out of 10 papers). There is only one systematic review and meta-analysis, underscoring the need for high-level evidence research in surgical endodontics.

As previously mentioned, characteristic peaks in scientific production observed over the years coincide with research findings that have led to breakthroughs in the field. The discovery of MTA around 1995 prompted a more thorough examination of the material by the research community. Numerous publications explored its properties and potential clinical applications, resulting in a significant shift in clinical practice and establishing MTA as the gold standard in surgical endodontics. The success of MTA also sparked further research on calcium silicate cements, leading to another peak in scientific production and a change in current clinical practices. A corresponding shift in clinical practice was evident with research findings on instruments and devices. Magnification devices, ultrasonics, and piezo tips were found to enhance the predictability of procedures and have become essential components of clinicians’ armamentarium when performing surgical endodontics. In conclusion, this analysis confirms the interdependent relationship between research and clinical practice and highlights the major impact of certain discoveries prompting further qualitative and quantitative studies.

The retrieval of eligible studies from two basic databases represents a major strength of the present study, overcoming a significant limitation of bibliometric analysis that relies on a single database. The automatic removal process of duplicate records eliminates the potential for bias or human errors. The methodology used ensures the inclusion of relevant publications and the reproducibility of the findings. At the same time, there are some limitations in this bibliometric analysis. The ongoing evolution of the field of surgical endodontics as demonstrated by the continuous publication of scientific papers cannot be steadily followed but only captured in a specific time frame. Another limitation is that scientometric analysis is unable to compare and synthesize the available data to draw conclusions addressing questions aroused by the scientific community [11]. Finally, research work of great importance and quality may be overlooked and this may hinder the evolution of the scientific field.

This paper evaluates the characteristics and evolution of scientific knowledge in surgical endodontics using bibliometric analysis methods. In surgical endodontics, various areas have been explored over the years with a primary focus on materials used in clinical practice. In the last 10 years, apart from materials, research has shifted toward the exploration of innovative techniques that constitute evolving research fields in surgical endodontics. These techniques utilize cone-beam computed tomography and leverage its capabilities in emerging innovative tools, such as navigation systems and planning software. Nevertheless, the incorporation of standardized surgical protocols, along with clearly defined success criteria from high-level evidence-based research studies, should be utilized when evaluating prognostic factors in surgical endodontics.

## Data Availability

The authors confirm that the data supporting the findings of this study are available within the article.
